# A Tad pilus promotes the establishment and resistance of *Vibrio vulnificus* biofilms to mechanical clearance

**DOI:** 10.1038/s41522-018-0052-7

**Published:** 2018-04-23

**Authors:** Meng Pu, Dean Allistair Rowe-Magnus

**Affiliations:** 1 Department of Molecular and Cellular Biochemistry, Indiana University of Bloomington, Bloomington, IN USA; 2 Department of Biology, Indiana University Bloomington, Bloomington, IN USA

## Abstract

*Vibrio vulnificus* is autochthonous to estuaries and warm coastal waters. Infection occurs via open wounds or ingestion, where its asymptomatic colonization of seafood, most infamously oysters, provides a gateway into the human food chain. Colonization begins with initial surface contact, which is often mediated by bacterial surface appendages called pili. Type IV Tad pili are widely distributed in the Vibrionaceae, but evidence for a physiological role for these structures is scant. The *V. vulnificus* genome codes for three distinct *tad* loci. Recently, a positive correlation was demonstrated between the expression of *tad-3* and the phenotypes of a *V. vulnificus* descendent (NT) that exhibited increased biofilm formation, auto-aggregation, and oyster colonization relative to its parent. However, the mechanism by which *tad* pilus expression promoted these phenotypes was not determined. Here, we show that deletion of the *tad* pilin gene (*flp*) altered the near-surface motility profile of NT cells from high curvature, orbital retracing patterns characteristic of cells actively probing the surface to low curvature traces indicative of wandering and diminished bacteria–surface interactions. The NT *flp* pilin mutant also exhibited decreased initial surface attachment, attenuated auto-aggregation and formed fragile biofilms that disintegrated under hydrodynamic flow. Thus, the *tad-3* locus, designated *iam*, promoted *i*nitial surface attachment, *a*uto-aggregation and resistance to *m*echanical clearance of *V. vulnificus* biofilms. The prevalence of *tad* loci in the Vibrionaceae suggests that they may play equally important roles in other family members.

## Bacterial pili: “Hairs” that promote attachment

One type of the hair-like appendages called pili on the surface of *Vibrio vulnificus* bacteria promote the formation of resistant biofilms. *Vibrio vulnificus* inhabits estuaries and warm coastal waters. It typically infects humans by contact with open wounds or ingestion, most notably by eating infected oysters. Infection can cause gastroenteritis, wounds that will not heal, sepsis and death. Dean Rowe-Magnus and Meng Pu at Indiana University, USA, used gene deletion experiments to reveal the importance of a specific gene required to create one category of pili. In the absence of the gene the ability of the bacterial cells to explore, attach to and colonize surfaces was significantly diminished. The pili also promote bacterial aggregation into biofilms and make the biofilms resistant to disruption by the mechanical action of fluid flow.

## Introduction

Biofilms provide a protective environment for growth and, in nature, the majority of microorganisms live within complex biofilm communities.^1^ For many bacteria, Type IV pili (T4P) have been shown to play an important role in the switch from a free-swimming, planktonic lifestyle to a surface-attached, sessile existence by enhancing initial surface association and subsequent micro-colony formation.^2,3^ The pilin subunits that comprise T4P are synthesized as prepilins that are then processed prior to incorporation into the pilin structure. Prepilins are classified into two types (A and B) based on their characteristics and the systems used for their export and assembly.^4,5^ B-type prepilins can be further subdivided and the Flp subtype is associated with the tight adherence (*tad*) pilus locus.^6–8^

The *tad* locus was first reported as being required for biofilm formation by the oral pathogen, *Actinobacillus actinomycetemcomitans* and homologous loci were subsequently identified in several other pathogens.^8^ Lesions in the *tad* locus decreased biofilm formation or caused attenuation in several of these species,^9,10^ leading to the locus being dubbed a widespread colonization island due to its requirement for colonization or virulence by these pathogens and its conservation in diverse archaeal and bacterial species.^8,11^

The Vibrionacea includes three main human pathogens: *Vibrio cholerae*, *V. parahaemolyticus*, and *V. vulnificus.*^12^ *Vibrio cholerae*, which causes millions of cholera cases and 170,000 deaths each year,^13^ uses the A-type mannose-sensitive hemagglutinin (MSHA) and chitin-regulated competence (ChiRP) pili to attach to abiotic and chitinous surfaces, respectively.^14,15^ The B-type toxin coregulated pilus is essential for intestinal colonization and pathogenesis.^16^ The MSHA and ChiRP pili have been shown to play similar roles in *V. parahaemolyticus,*^17,18^ a major cause of gastroenteritis, and *V. vulnificus,*^19–21^ which has the highest death rate (>35%) and economic burden per case (>$3,000,000) of any foodborne pathogen in the United States.^22^ Homologs of the *tad* locus are widely distributed in the Vibrionaceae and many *Vibrio* genomes encoded multiple *tad* loci;^8,23^ however, a physiological function had not be described for a Tad pilus for any family member. The *V. vulnificus* genome codes for three distinct *tad* loci (*tad-1*, *tad-2*, and *tad-3*). We recently showed that *tad-3* expression contributed to the increased biofilm formation and oyster colonization phenotypes of a *V. vulnificus* descendent (NT) relative to its parent. However, the mechanism by which *tad-3* pilus expression promoted these phenotypes was not known. Here, we show that its expression promoted initial bacterial surface attachment and auto-aggregation, and increased the resistance of *V. vulnificus* biofilms to mechanical clearance—phenotypes associated with efficient niche (oyster) colonization.^20,23,24^

## Results

### *tad-3* expression supports the formation of large NT aggregates

**Fig. 1:**
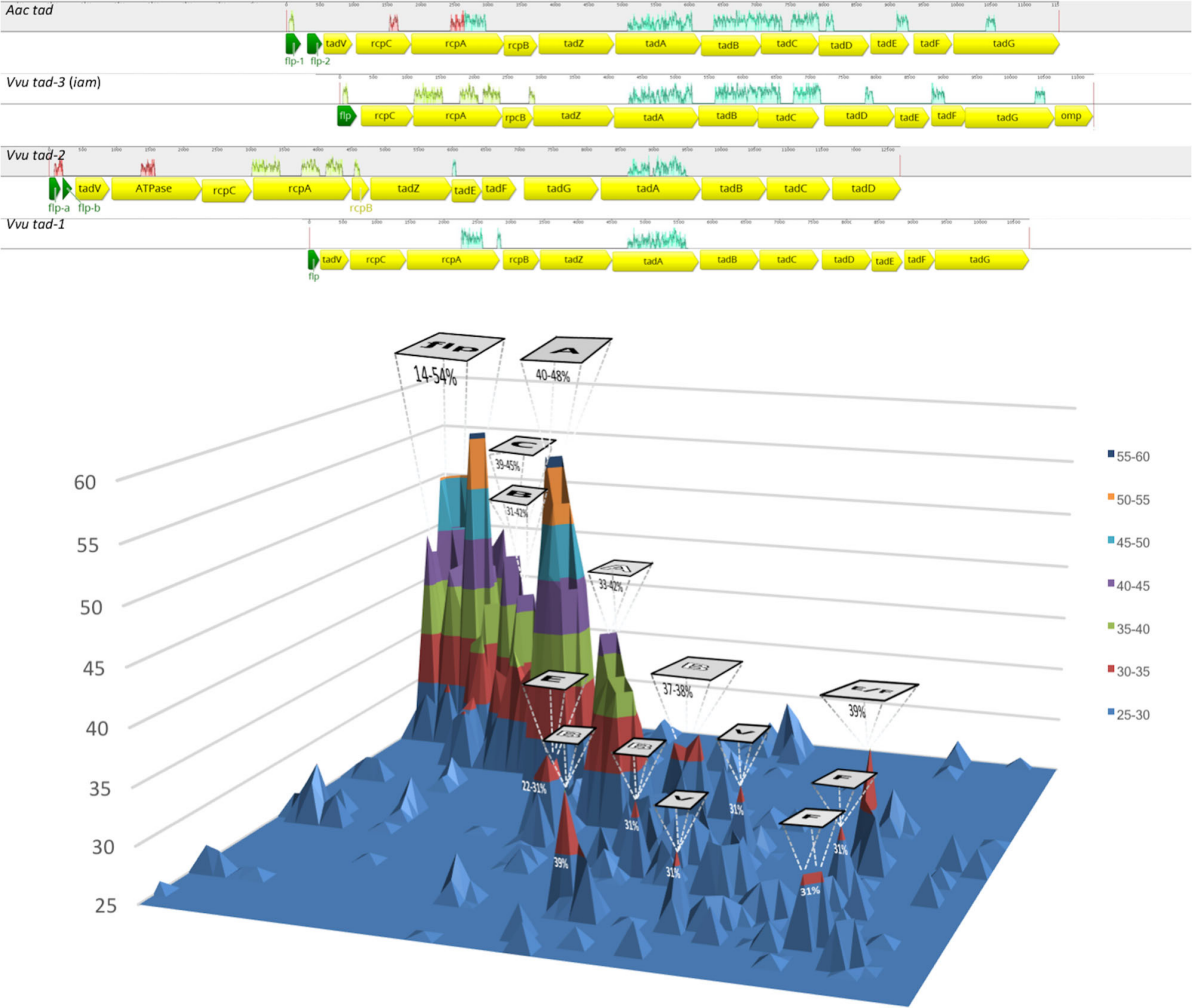
Homology of the *tad* loci of *V. vulnificus*. Top, mauve alignment of the *tad* locus of *A. actinomycetemcomitans* and the *tad-1, tad-2*, and *tad-3* loci of *V. vulnificus*. The loci are shown arranged centered on the conserved *tadA* gene. Coding sequences are yellow and *flp* pilin genes are green. Highly conserved regions between the loci are denoted by colored traces above the ORFs. The pilin genes of the *tad-2* locus are labeled *a* and *b*. The length of each locus is shown at the top of each panel. Bottom, distance matrix surface plot of the *tad* genes of the loci above. Homologous gene clusters form peaks, the height of which indicates the percent of homology. Only clusters with >25% homology are shown (legend on the right). *flp* and *tad* genes are shown in black lettering and *rpc* genes are in white lettering

The *V. vulnificus* genome codes for three *tad* loci, designated *tad-1*, *tad-2*, and *tad-3*, that are homologs of the *A. actinomycetemcomitans tad* locus^11,23^ (Fig. 1). The *tadABC*, *rcpAB*, *flp*, *tadV*, and *tadEF* genes shared the highest homology (31–56%) among the *tad* loci.

**Fig. 2:**
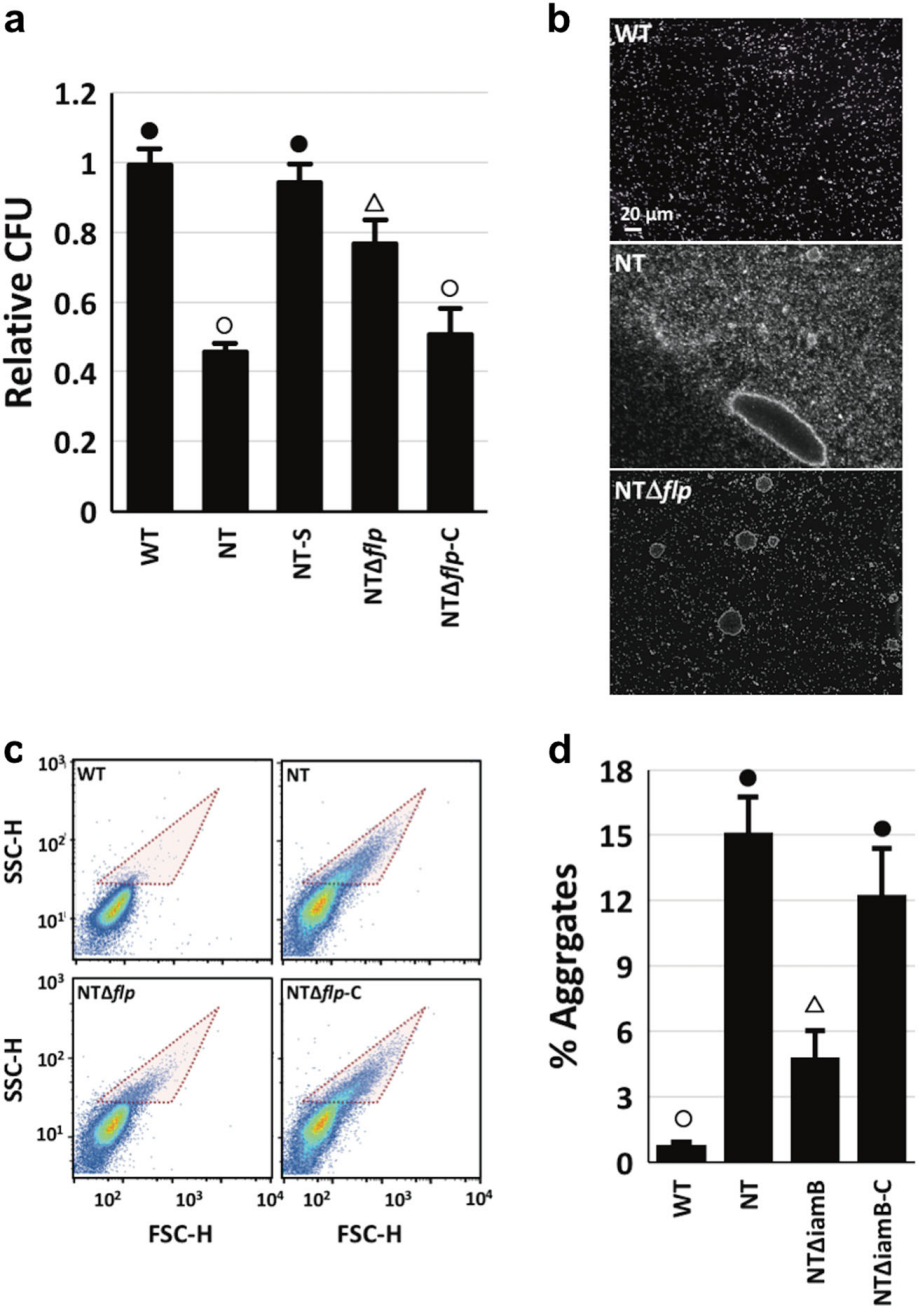
The Tad pilus enhances *V. vulnificus* auto-aggregation. **a** Relative CFU (post/pre 50 μm filtration) for the indicated strains. **b** Dark-field images (×20) of WT (top), NT (middle), and NTΔ*flp* cells grown in liquid culture. Scale bar is in the lower left. **c** FACS analysis of aggregates (pink shaded area) in 50 μm filtrates of the indicated strains. **d** Quantitation of aggregation in **c**. Statistically significant differences among the samples (*p* < 0.005) as determined by one-way analysis of variance (ANOVA) are indicated by different symbols above each bar

Unlike WT, NT grew as visible clumps (Fig. S1). To gain insight into the level at which *tad-3* contributed to this aggregation phenotype, bacterial counts in 50-μm filtrates (CFU_f_) relative to the starting culture (CFU_s_) were determined for the WT, NT, NT*Δflp*, and complemented (NT*Δflp*-C) strains (cultures with an optical density at 600 nm of 0.1–0.15 were used). The CFU_f/s_ of WT cultures was 0.992, indicating that the majority of cells in the starting culture were recovered in the 50-μm filtrate (Fig. 2a). Over half of NT cells were removed following the filtration step (CFU_f/s_ = 0.456), suggesting that many of the cells formed aggregates too large to traverse the filter. The CFU_f/s_ of NT cultures that were vortexed to shear surface appendages such as pili and flagella (NT-S) was 0.942, similar to WT. Deletion of *flp* (NT*Δflp*) increased the number of cells in the filtrate relative to NT (CFU_f/s_ = 0.766) and complementation of NT*Δflp* with a plasmid-borne copy of *flp* decreased the CFU_f/s_ to 0.506. Imaging of the starting cultures suggested that WT existed largely as single cells, while NT could self-associate into massive aggregates >100 μm in diameter (Fig. 2b). Large aggregates (>20 μm) were clearly visible for NT*Δflp*, but those >50 μm in size were in lower abundance relative to NT. Together, these results suggested that pilin production promoted *V. vulnificus* auto-aggregation.

FACS analysis of 50-μm filtrates was used to more accurately measure size distribution in the cell population. Greater heterogeneity was observed for NT cells than WT cells (Fig. 2c). WT bacteria primarily existed as single cells (0.8% aggregates), while 15% of NT cells formed larger aggregates (Fig. 2d). Deletion of *flp* (NT∆*flp*) decreased the percentage of aggregates to 4.8% and this could be complemented following *flp* expression from a plasmid (12% aggregates for NT∆*flp*-C). These results suggested that *flp*, and by extension the Tad pilus, contributed to the increased auto-aggregation phenotype of NT.

### *tad-3* expression contributes to rugose colony development

**Fig. 3:**
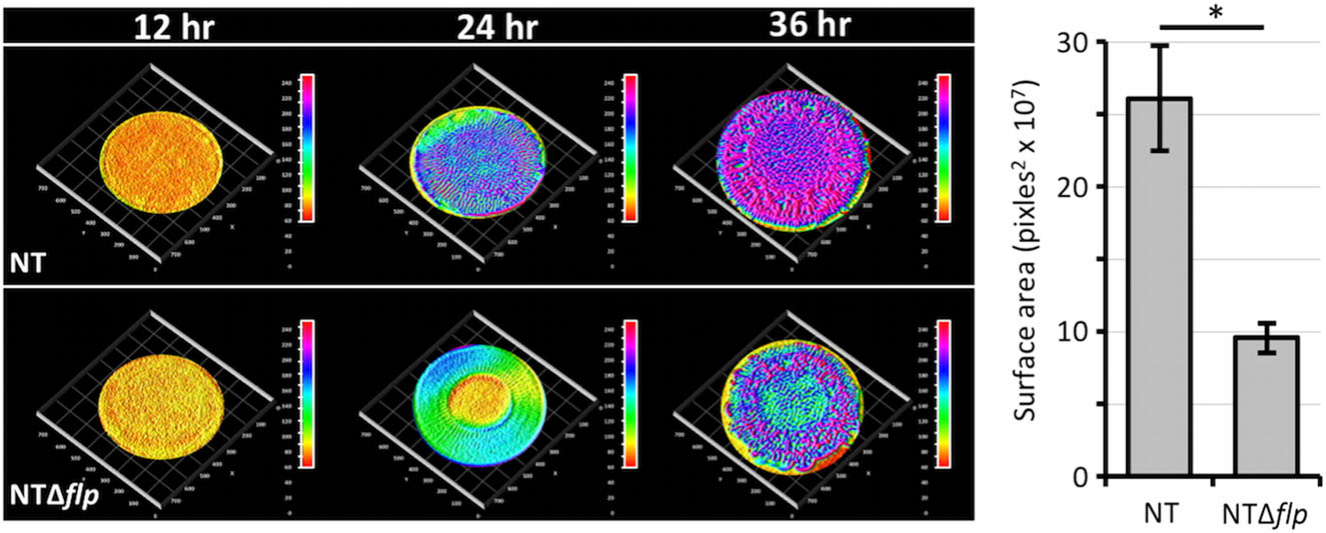
The Flp pilin contributes to rugose colony development. Rugose colony development of NT and NTΔ*flp* cells expressing DcpA was monitored over 36 h. Elevation map, minimum (red) and maximum (purple). A representative image of samples done in triplicate is shown. To the right is a plot of the calculated surface area of the indicated strains. Statistically significant differences between the samples (*p < 0.01) was determined by the Student’s *t* test

We previously demonstrated that elevated intracellular c-di-GMP concentrations induce rugose colony development in *V. vulnificus.*^25,26^ To determine if *tad-3* contributed to the rugose phenotype, the diguanylate cyclase DcpA was expressed in NT and NTΔ*flp* cells to increase intracellular c-di-GMP levels. Rugosity developed in both backgrounds but with different temporal and apical dynamics (Fig. 3). Colonies remained flat and featureless in both backgrounds over the first 12 h. By 24 h, the dramatic surface undulations that are a hallmark of rugose colony development were clearly visible for NT, while vertical expansion of NTΔ*flp* colonies was less evident. Notably, NT exhibited a well-developed rugose phenotype by 36 h. Comparatively, rugose colony formation by NTΔ*flp* was both slow and diminished. Although some surface corrugation was observed, rugosity failed to fully develop after 36 h (total surface area was 2.5-fold lower than NT). This suggested that a functional Tad pilus contributed to the full and timely development of the rugose phenotype.

### *tad-3* expression mediates initial bacterial surface interactions

**Fig. 4:**
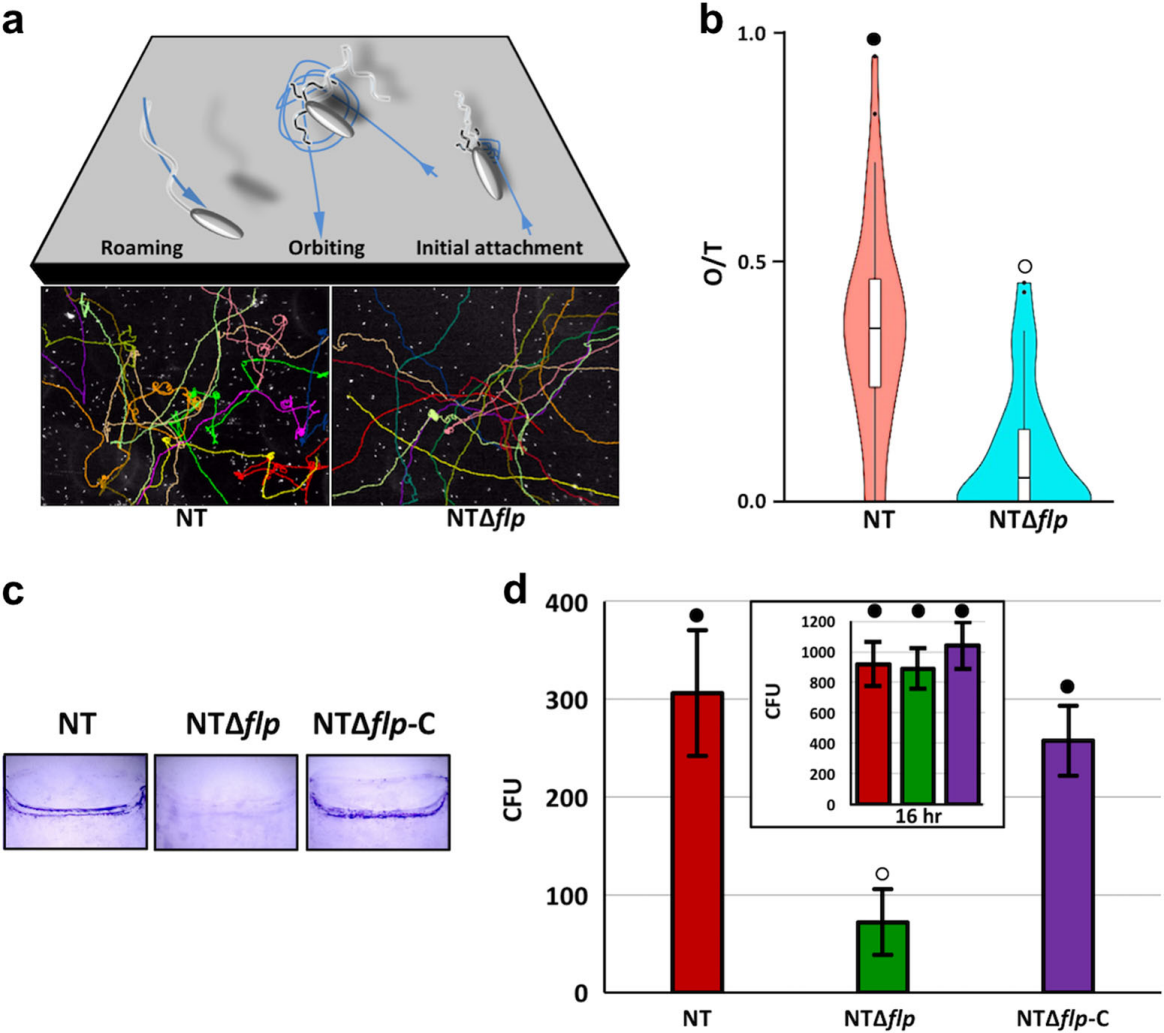
The Tad pilus promotes initial surface attachment by *V. vulnificus*. **a** Schematic of anticipated movements (above) and representative colored traces (below) of NT and NTΔ*flp* cells. **b** Violin plot of orbiting frequency for NT, NT∆*flp*, and complemented cells. Orbiting-to-total (O/T) ratio is the number of frames spent in a high curvature pattern within a 10 × 10 μm^2^ area/total number of frames. Horizontal bars mark the mean for each sample. **c** Initial surface attachment (2 h) to coverslips by the indicated strains. Adherent bacteria were detected by CV staining. **d** Quantitation of adherent bacteria in **c**. Inset, cell attachment after 16 h of incubation. Statistically significant differences among the samples (*p* < 0.001) as determined by one-way analysis of variance (ANOVA) are indicated by different symbols above each bar

*Vibrio cholerae* has been shown to exhibit two distinct motility behaviors, termed roaming and orbiting, when approaching a surface for initial attachment.^14^ Roaming is defined by low curvature traces that result from weak contacts between MshA pili and the surface, while orbiting is exemplified by high curvature, circular tracks that retrace over the same area and result from strong pili-surface contacts.^27^ The majority of *V. cholerae* cells that eventually attach were shown to originate from the orbiting cell population. To determine if the Tad pilus impacted the near-surface motility behavior of *V. vulnificus*, the movement of NT and NT*Δflp* cells was tracked and the traces were analyzed (Fig. 4a, b). The time cells spent orbiting/retracing an area was nearly fourfold higher for NT than NT*Δflp* (mean of 0.37 compared to 0.10, *p* < 6 × 10^−9^). These results suggested that the deletion of *flp* skewed the near-surface trajectory of *V. vulnificus* from orbiting towards roaming. We surmised that this negative effect on initial bacteria–surface interactions might also inhibit initial surface attachment. Indeed, NT readily colonized a coverslip surface in a 2 h initial attachment assay, while virtually no attachment was detected for NT*Δflp* (Fig. 4c, d). Initial surface adherence was recovered following complementation with *flp* (NT*Δflp*-C). However, when bacteria were allowed to adhere for 16 h, comparable levels of surface colonization were observed for NT and NT*Δflp* (Fig. Fig. 4d, inset), indicating that NT*Δflp* was eventually able to colonize given a prolonged contact time. When adherent NT*Δflp* cells were re-inoculated into fresh media, they again attached poorly over the initial 2 h contact window. This suggested that the ability of NT*Δflp* to eventually attach was likely not due to the emergence of a mutant that suppressed the initial attachment defect. Together, these results suggested that the Tad pilus plays an important role during the initial attachment phase when a surface is being probed for colonization but its loss did not prevent eventual surface association in a closed static system.

### *tad-3* expression promotes resistance of *V. vulnificus* biofilms to mechanical clearance

**Fig. 5:**
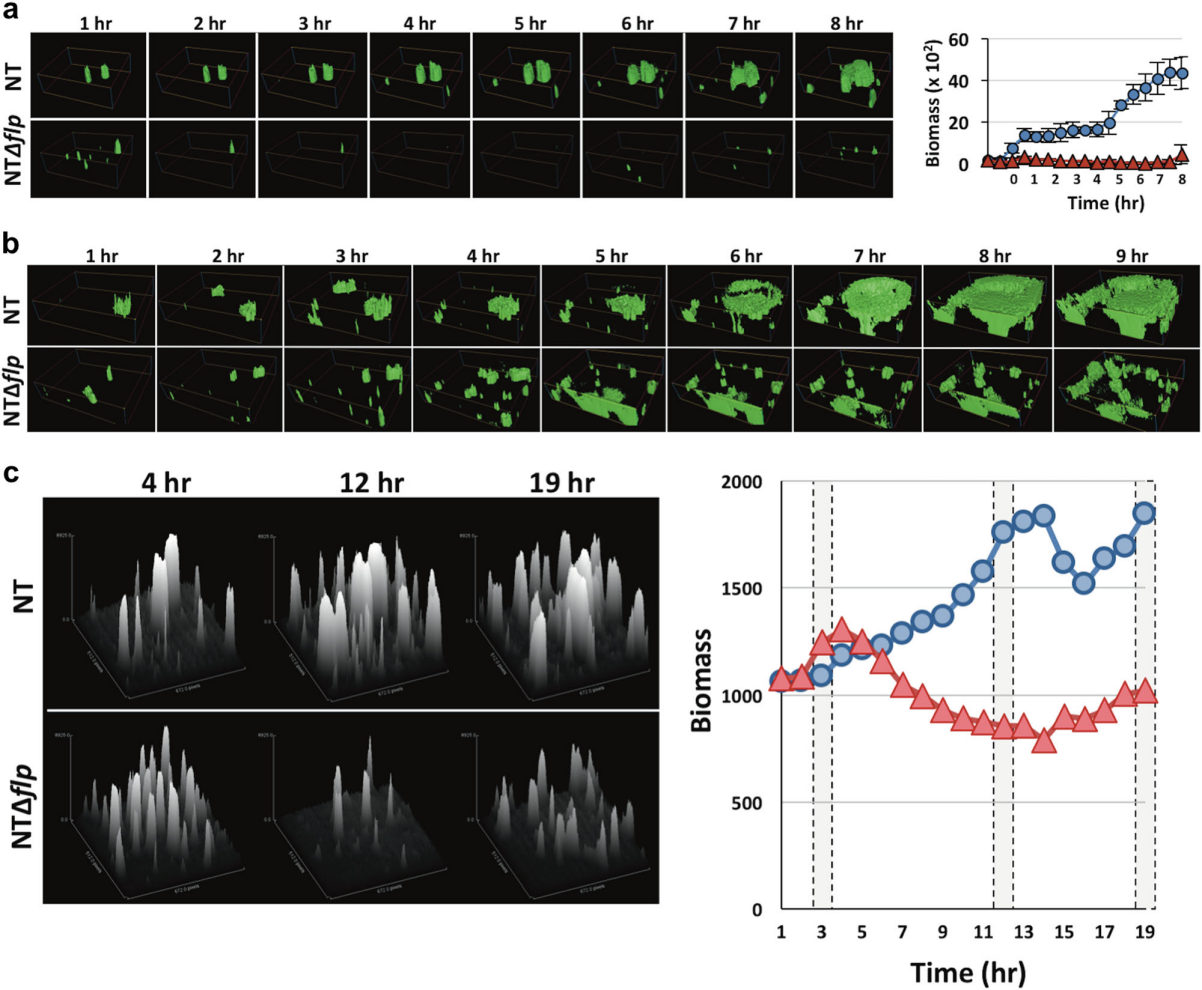
The Tad pilus promotes biofilm maturation under hydrodynamic flow. **a** Mid-exponential NT (top) and NTΔ*flp* (bottom) cells expressing *gfp* were inoculated in micro-fluidic flow-cell chambers and biofilm development was monitored in micro-fluidic flow cells under flow (3 ml min^−1^). Image stacks were captured hourly. Right, quantitation of the GFP signal in each stack over the time course. Blue, NT; red, NTΔ*flp*. **b** Biofilm formation by the same strains (NT, top panel; NT∆*flp*, bottom panel) was monitored in micro-fluidic flow cells under low-flow conditions (0.75 ml min^−1^). **c** Biofilms of *gfp*-expressing NT (top) and NT∆*flp* (bottom) cells were established under low-flow conditions and allowed to develop for 3 h. Flow was then increased to 3 ml min^−1^ and biofilm fate was tracked hourly for another 16 h. The biomass of image stacks from the 3, 12, and 19 h time points was determined and plotted in the adjacent graph (blue, NT; red, NT∆*flp*)

To better mimic environmental conditions, biofilm formation by NT and NT*Δflp* strains expressing *gfp* was monitored under hydrodynamic flow. The strains were seeded into separate micro-fluidic flow-cell chambers and biofilm development was tracked hourly. NT readily established micro-colonies at moderate flow (3 ml min^−1^) and progressed to macro-colony formation (Fig. 5a). Macro-colonies expanded in place or following contact with bacterial aggregates carried by the flow (Video S1). The eventual outcome was a well-developed and structured biofilm. Conversely, biofilm formation by NT*Δflp* was severely hampered. NT*Δflp* micro-colonies that were initially established during chamber inoculation were displaced under flow (3 ml min^−1^), preventing progression to a mature biofilm. Despite this, an appreciable NT*Δflp* biofilm could eventually be established under low flow (0.75 ml min^−1^, Fig. 5b). However, NT*Δflp* still required considerably more time than NT to securely attach to the surface and achieve a substantial biomass. The resulting NT*Δflp* biofilm was also fragile, since increasing the flow to 3 ml min^−1^ led to its disintegration, whereas the NT biofilm remained intact. However, the *flp* mutant could be incorporated into maturing biofilms that were resistant to high flow rates (5 ml min^−1^) when inoculated in mixed culture with NT cells (Figure S2). These results suggested that the Tad pilin stabilized the maturing biofilm. The *tad* locus was therefore designated *iam* for its critical role in *i*nitial attachment, *a*uto-aggregation and resistance to *m*echanical clearance of *V. vulnificus* biofilms.

## Discussion

T4P serve diverse functions in Gram-negative bacteria.^3^ They are known to mediate biofilm formation, surface and twitching motility,^28^ natural transformation,^29^ transduction,^30^ and host–cell interactions.^5^ We previously isolated a *V*. *vulnificus* descendent (NT) that exhibited an increase in biofilm formation and oyster colonization relative to the parental strain.^23^ We found that a *tad* pilus locus, one of three encoded in the genome, was upregulated in NT and demonstrated that its expression promoted the key biofilm-related phenotypes of initial attachment, auto-aggregation, and resistance to mechanical clearance. We have designated this locus *iam* (previously *tad-3*) to reflect its impact of *V. vulnificus* physiology when expressed and to distinguish it from the two other *tad* loci in the genome, which may affect different aspects of *V. vulnificus* biology.

Pilin-dependent initial surface attachment is an important stage of biofilm formation. It was recently demonstrated in *V. cholerae* that flagellar-driven counter rotation of the bacterial cell body periodically brings MSHA pili into contact with a nearby surface.^14^ Strong pili-surface interactions extended the linger time of a cell in an area, resulting in an orbital motion pattern. Notably, only orbiting cells could irreversibly attach to a surface and form micro-colonies. We observed that an *flp* pilin mutant (NTΔ*flp*) spent less time orbiting and probing the surface than NT, suggesting that deletion of *flp* impaired the near-surface motility behavior of *V. vulnificus*. As a consequence, initial surface attachment by NTΔ*flp* was attenuated relative to NT. This was similar to the motility and biofilm phenotypes observed for a *V. cholerae mshA* pilin mutant^14,27^ and supports a role for the Iam pilus in establishing initial bacteria–surface contact in *V. vulnificus*.

**Fig. 6:**
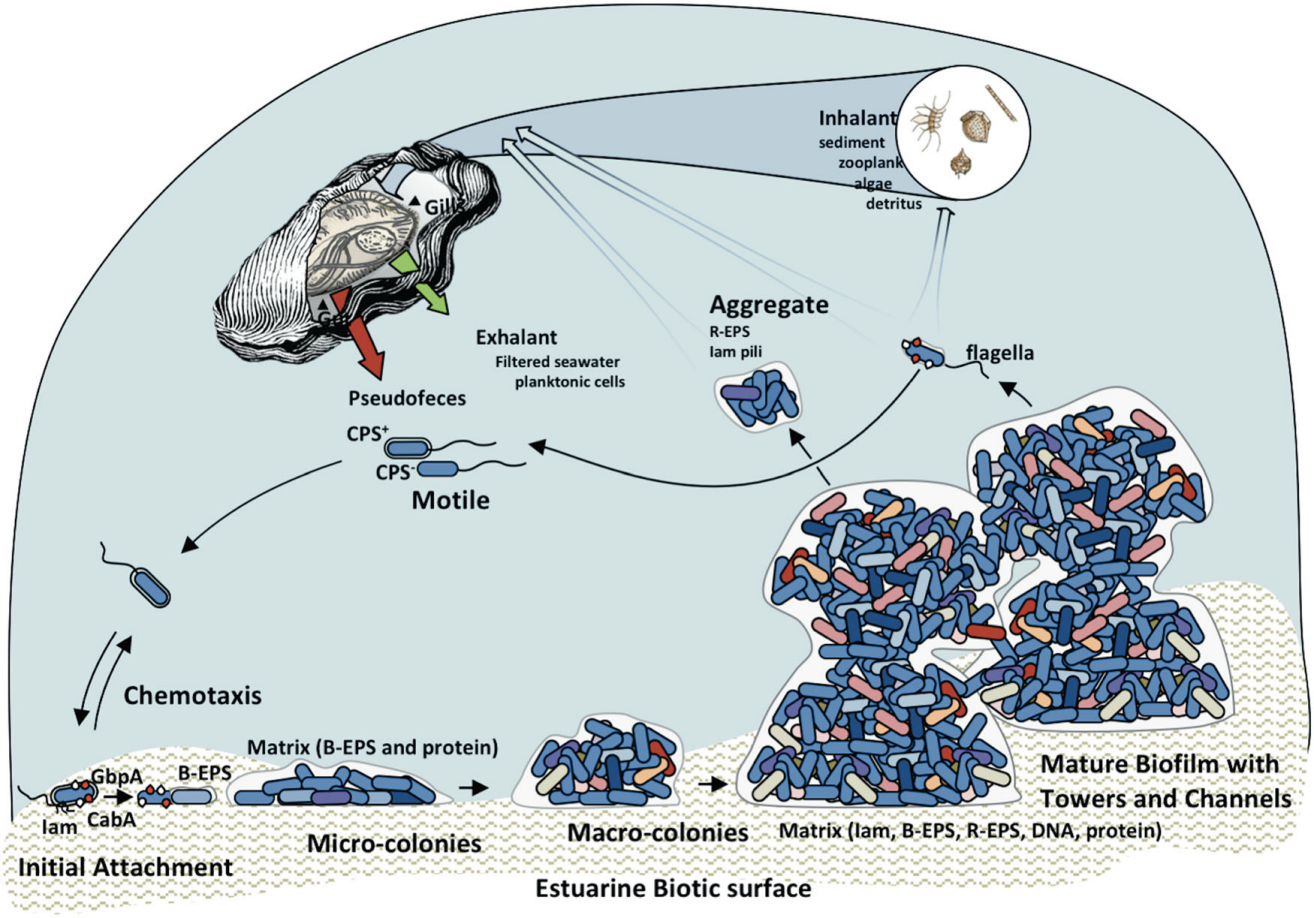
Model for the colonization of environmental surfaces by *V. vulnificus*. Motile bacteria powered by flagella may (+) or may not (−) express CPS. Chemotactic and other responses guide cell movement. Initial contact with a biotic surface is aided by adherence factors such the Iam pilus. The calcium and chitin binding factors, CabA and GbpA (white and red teardrops), as well as the MSHA and ChiRP pili likely play important roles in binding to specific surfaces such as crustacean exoskeletons and zooplankton. The pilus, in conjunction with the *brp*-encoded EPS (B-EPS) promotes irreversible attachment and the establishment of micro-colonies. Subsequent macro-colony and full biofilm development is suspected to involve the coordinated input of additional factors, including the *rbd*-encoded EPS (R-EPS) and DNA. Environmental conditions and host responses give rise to heterogeneous populations within the mature biofilm (growing/dividing (light blue), stationary (medium blue), dead (dark blue), pathogenic (red), competent (pink), VBNC (purple), lysing (orange), etc. The triggering of biofilm dissolution leads to the release of bacterial aggregates and individuals that can re-colonize a similar or different surface (sediment, zooplankton, algae, detritus) and be taken up as oyster inhalant. Food particles are digested and pseudofeces is ejected. Planktonic cells are poorly retained and typically pass through as part of the filtered exhalant. Larger aggregates are more efficiently retained and drive host colonization

*Vibrio vulnificus* biofilm formation is multifactorial (Fig. 6). Flagellar motility propels bacteria toward a surface. Capsular polysaccharide (CPS) production is essential for virulence but inhibits biofilm formation.^31^ The Iam pilus plays a key role during the initial attachment stage by prolonging the bacteria–surface contact time. The MSHA and ChiRP pili may also participate, as mutations in either can negatively impact biofilm formation in certain conditions.^14,19,20,32^ Irreversible surface attachment is dependent on expression of the c-di-GMP-regulated *brp-*encoded EPS.^25,33^ GbpA and CabA^34–36^ are key matrix-associated proteins that contribute to *V. vulnificus* biofilm development. GbpA mediates attachment to chitinous substrates and CabA is a calcium-dependent extracellular protein required for biofilm maturation, rather than initial attachment.^34^ Micro-colony and macro-colony formation ensues and the Iam pilus, in conjunction with CabA, functions to maintain the integrity of the developing biofilm. Motile single cells or aggregates may be released from the mature biofilm. Bivalves such as oysters have the ability to size-select particles for ingestion—the retention of particles of <5 µm is poor (<20%) for most bivalves but particles >5 µm are captured from the inhalant with nearly 100% efficiency.^37^ Planktonic *Vibrio* species are approximately 1 × 2 μm^2^. Thus, the incorporation of bacteria into marine aggregates via self-association, attachment to sediment, zooplankton, algae, or detritus can increase their retention within feeding bivalves, leading to the potential accumulation of pathogenic microorganisms.^24^ *Vibrio vulnificus*, perhaps best known for colonizing oysters, can reach densities of 10^5^–10^6^ bacteria g^−1^ in summer months.^38–40^ We have shown that NT, which exhibited an enhanced auto-aggregation phenotype, was more readily captured by suspension-feeding oysters than the planktonic parental strain.^23^ Oysters are also able to pump and process large volumes of the surrounding water column, up to 3 L h^−1^ for a single oyster.^41^ The ability of *V. vulnificus* biofilms to withstand mechanical clearance by the fluid flow of feeding oysters that would otherwise purge them from a surface is fundamental for successful host colonization in the environment.^42^ We observed that NT biofilms resisted mechanical clearance, whereas NTΔ*flp* biofilms quickly disintegrated and had difficulty re-establishing under flow. The ramifications of *flp* deletion, diminished and fragile biofilms that were readily cleared under hydrodynamic flow, and the marked reduction in oyster colonization by a NT∆*flp* mutant^23^ underscores the importance of the Iam pilus in niche colonization. The prevalence of *tad* loci in the Vibrionaceae^8,23^ suggests that they may play a similar role in other family members.

## Materials and methods

### Strains and growth conditions

Media were purchased from BD Difco. Antibiotics and additives were purchased from Sigma and used as follows: ampicillin (Ap), 100 µg ml^−1^; gentamycin (Gm), 10 (*E. coli*) or 35 µg ml^−1^ (*V. vulnificus*); rifampicin (Rf), 100 µg ml^−1^; isopropyl-β-d-thiogalactoside, 100 µM; l-arabinose (l-ara), 0.1%. An Rf-resistant isolate of *V. vulnificus* ATCC27562 was used as the parental strain and *E. coli* S17.1λπ was used for conjugation to *V. vulnificus*. *Vibrio vulnificus* NT and NT∆*flp* were previously described.^23^

### Rugose colony imaging

NT and NT∆*flp* strains carrying pBAD24T-*dcpA*^25,43^ were grown in LB Gm overnight at 30°C with shaking. Cultures were then diluted 1:200 in fresh LB Gm l-ara and grown to mid-log phase. A 2 µl aliquot was spotted onto LB Gm l-ara plates and incubated at 30°C for 36 h and photographed with an Axio-Cam MRc5 (Zeiss) digital camera connected to a M60 stereo microscope (Leica). A fixed radius was used to determine the surface area for colonies with the SP package^44^ in R.^45^ Corresponding 3D elevation maps were created with ImageJ.^46^ A representative image of samples done in triplicate is shown.

### Flow cytometry

NT and NTΔ*flp* strains expressing *gfp*^23^ were grown in LBS (LB containing 2% NaCl) medium overnight at 30 °C. The cells were passed over a 50-μm filter and flow cytometry of the filtrates was performed on a FACSCalibur flow cytometer (BD Biosciences) equipped with a blue laser (*λ*_ex_ = 488 nm) and a band pass filter measuring green fluorescence (FL1; 530/30 nm). The sample flow rate was set at “low” (12 ± 3 μl min^−1^) and 50,000 events were recorded within a preset gate defining the viable cell population. Analyses were performed on biological triplicates and the data was analyzed using FlowJo X (Tree Star).

### Motility tracking

The motility of early-exponential (OD_600_ of 0.1) *V. vulnificus* cells was recorded by dark-field microscopy on an Olympus IX83 microscope using a ×20 ELWD objective. A stack of at least 150 frames (100 ms exposure time) was recorded for each sample and the movement of 15 randomly sampled motile cells per stack was traced using the MTrackJ^47^ plugin in Comstat.^48^ The number of frames a cell spent orbiting (O) within a 10 × 10 μm^2^ area was divided by the total number of frames (T) to give O/T. Data from three stacks of three biological replicates were analyzed for each strain. Traces shown are from a single experiment. Violin plots were created with ggplot2.^49^

### Biofilm development in micro-fluidic chambers

Polydimethylsiloxane qglass flow-cell devices containing eight 40 × 5 × 1 mm^3^ chambers were fabricated as previously described^50^ (see Supplementary Information for details). The chambers were sterilized by sequential treatment with 50 ml each of 3% H_2_O_2_, sterile H_2_O, and LBS prior to inoculation. Mid-log *gfp*-expressing NT and NTΔ*flp* cultures (OD_600_ of 0.1) were seeded into separate flow-cell chambers. For mixed culture experiments, NTΔ*flp* cells expressing *td-Tomato* were used to distinguish them from *gfp*-expressing NT population. Initial attachment (no flow) proceeded for 20 min followed by a flow rate of 0.75 ml min^−1^ (low), 3 ml min^−1^ (normal), or 5 ml min^−1^ (high) where indicated. Biofilm images and z-stacks (20 × 1 μm^2^ slices) were captured with an Olympus IX83 microscope using a UPLSAPO ×40 silicon oil immersion objective (NA 1.25, WD 0.3 mm). Quantitative analysis to determine biomass was performed using cellSense (Olympus) and Comstat.^48^ Data from three biological replicates were analyzed for each strain. Images presented are from a single representative experiment.

### Genomic and statistical analyses

The *tad* pilus sequences were searched against the Vibrionales (NCBI and Uniprot databases) using BLAST^51^ (*E*-value cut-off of <9.4e^−152^). Conserved genomic regions were detected using the Mauve genome aligner.^52^ The distance matrix surface plot for a translation alignment of the *tad* locus from *A. actinomycetemcomitans* and the three *V. vulnificus tad* loci was created in Excel (Microsoft). Statistical analyses for all plots were determined by one-way analysis of variance or Student’s *t* test where indicated.

### Data availability

All data are available via the npj website.

## Electronic supplementary material

SI Materials & Methods

Video S1

Figure S1

Figure S2
