# Supplementary material for: A Tad pilus promotes the establishment and resistance of Vibrio vulnificus biofilms to mechanical clearance
Source: NPJ Biofilms Microbiomes. 2018 Apr 23;4:10. doi: 10.1038/s41522-018-0052-7 (PMC5913241; doi:10.1038/s41522-018-0052-7)
Supplement: Supplementary file 1 — SI Materials & Methods [file 41522_2018_52_MOESM1_ESM.docx]

**A Tad pilus promotes the establishment and resistance of *Vibrio vulnificus* biofilms to mechanical clearance – Supplementary Information**

Meng Pu^b^ and Dean A. Rowe-Magnus^a,b^

^a^Department of Biology and ^b^Molecular and Cellular Biochemistry, Indiana University Bloomington, IN

**Materials and Methods**

**Biofilm development in microfluidic chambers**

Polydimethylsiloxane (PDMS)-glass microfluidic devices composed of a layer of PDMS and a layer of glass were fabricated as previously described^1^ with the following modifications. A brass master mold (fabricated at the machining facilities in the Physics Department at Indiana University Bloomington) containing eight channels (40 x 5 x 1 mm) with inlet and outlet access ports was filled with 15 ml of uncured PDMS Sylgard 184 (Dow Chemicals), the assembly was degassed under vacuum for 30 min and then cured for 8 hr at 65°C. The inlet and outlet access holes for each chamber were punched. The microfluidic device and a No. 1.5 glass coverslip (7.5 x 5.3 cm) were plasma cleaned (PDC-32G, Harrick Plasma), brought into direct contact with each other and cured at 65 °C for 1 hr to complete the bonding process. Flow cell chambers were sterilized by treatment with 50 ml of 3% H_2_O_2_ solution followed by equilibration with 50 ml of sterile H_2_0 and 50 ml of LBS prior to inoculation. Mid-log *gfp*-expressing NT and NTΔ*flp* cultures were adjusted to an OD_600_ of 0.1 and each was seeded into separate chambers of the microfluidic device. For mixed culture experiments, NTΔ*flp* cells expressing *td-Tomato* were used to distinguish them from *gfp*-expressing NT population. Initial attachment was performed in the absence of flow for 20 min followed by a constant flow rate of 3 ml min^-1^. Low flow was conducted at 0.75 ml min^-1^ and high flow rates were at 5 ml min^-1^. Biofilm images and z-stacks (20 x 1 micron slices) were captured with an Olympus IX83 microscope and a UPLSAPO

40X silicon oil immersion objective (NA 1.25, WD 0.3 mm). Quantitative analysis of image z-stacks to determine biomass was performed using cellSense (Olympus) and Comstat^2^. Data from three biological replicates were analyzed for each strain. Images presented are from a single representative experiment.

**References**

1. Williams, M. *et al.* Short-Stalked Prosthecomicrobium hirschii Cells Have a Caulobacter-Like Cell Cycle. *J Bacteriol* **198,** 1149–1159 (2016).

2. Heydorn, A. *et al.* Quantification of biofilm structures by the novel computer program COMSTAT. *Microbiology (Reading, Engl)* **146,** 2395–2407 (2000).

**Supporting Information Legends**

**Video S1. Building of a *V. vulnificus* biofilm.** Real-time tracking of aggregate formation during biofilm development. Large bacterial aggregates can be seen forming via the attachment of smaller aggregates. Differently colored arrows in A and B track different cluster-forming events while imaging the same area.
