# Supplementary figures and images for: A Tad pilus promotes the establishment and resistance of Vibrio vulnificus biofilms to mechanical clearance
Source: NPJ Biofilms Microbiomes. 2018 Apr 23;4:10. doi: 10.1038/s41522-018-0052-7 (PMC5913241; doi:10.1038/s41522-018-0052-7)

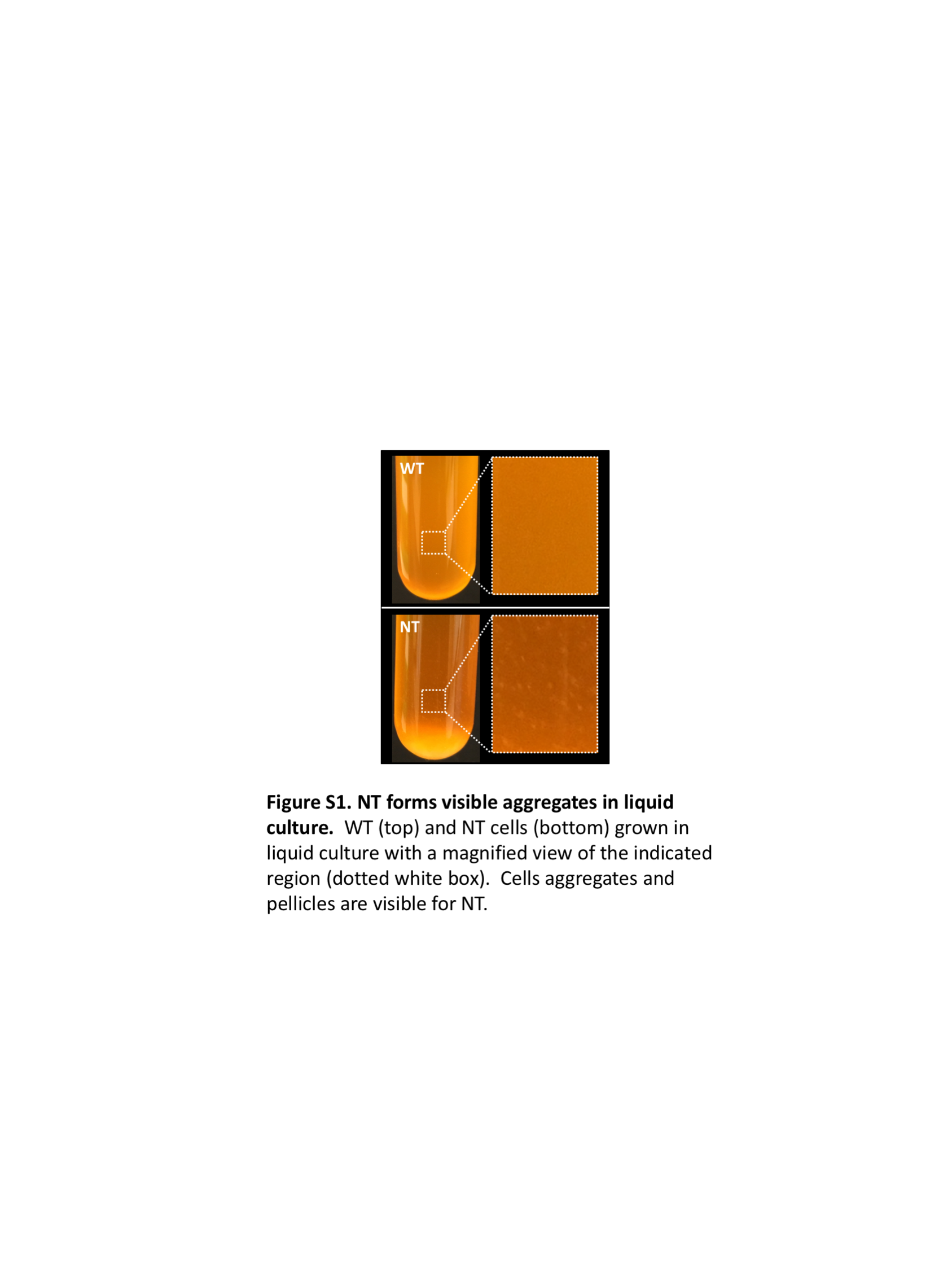

Supplement: Supplementary file 3 — Figure S1 [file 41522_2018_52_MOESM3_ESM.tif]
