# Supplementary material for: A Tad pilus promotes the establishment and resistance of Vibrio vulnificus biofilms to mechanical clearance
Source: NPJ Biofilms Microbiomes. 2018 Apr 23;4:10. doi: 10.1038/s41522-018-0052-7 (PMC5913241; doi:10.1038/s41522-018-0052-7)
Supplement: Supplementary file 4 — Figure S2 [file 41522_2018_52_MOESM4_ESM.pdf]

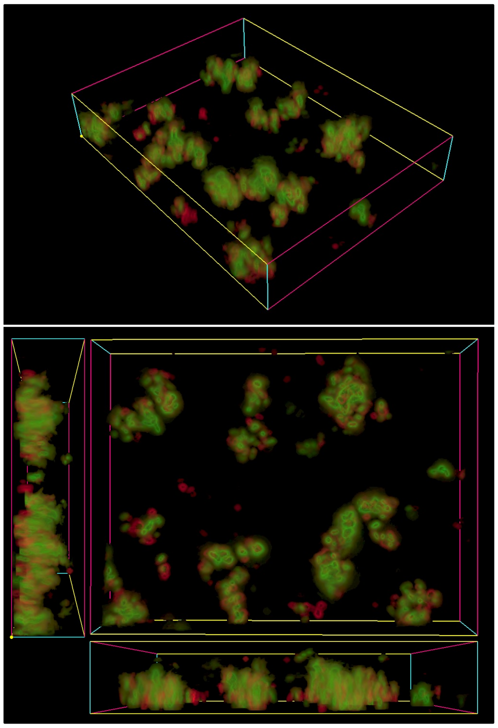

Figure S2. NTΔ*flp* can form stable mixed biofilms with NT. Biofilm formation by *gfp*-expressing NT and *tdTomato*-expressing NTΔ*flp* cells was monitored in micro-fluidic flow cells under high flow (5 ml min^{-1}) conditions. A 12-hour image stack of the mixed biofilm is shown. Top, angled view. Bottom, top-down and side views.
